# Supplementary material for: Indwelling Peritoneal Catheter for Ascites Management in a UK District General Hospital: A Cohort Study
Source: Healthcare (Basel). 2021 Sep 24;9(10):1254. doi: 10.3390/healthcare9101254 (PMC8536105; doi:10.3390/healthcare9101254)
Supplement: Supplementary file 1 [file healthcare-09-01254-s001.zip › healthcare-1374108-supplementary.pdf]

Indwelling peritoneal catheter (IPC) for malignant ascites guidelines	
**Document Type: Clinical Guideline**<br>**Clinical Lead: Avinash Aujayeb**<br>Author/s: Sarah Robinson, Leonie Armstrong, Katie Frew<br>**Business Unit/Directorate:** Acute and Emergency Medicine, Gastroenterology and General Surgery Approved by above consultants June 2018	**Date Ratified by Clinical Guidelines Group: 7 Aug 2018**<br>**Date of Issue:**<br>**Review Date: May 2021**<br>**Version: 1**

Relevant to: Northumbria Healthcare NHS Foundation Trust, Community Teams (Specialist palliative care teams, district nursing teams, General Practitioners), Ward Teams – NSECH/NTGH/WGH/HGH

Keywords –malignant ascites, indwelling peritoneal catheter

Criteria for use

This guideline is intended to guide clinicians for the referral and insertion of indwelling peritoneal catheters for malignant ascites

Important Notes

There are 2 types of IPCs on the market : Rocket® and Pleurex. Due to cost benefits, Northumbria Healthcare NHS Foundation Trust use the former.

Guidelines

1. **Indications**

   a. Any patient with malignant ascites

      (Special consideration is required for patients with gynaecological cancers as they often have major surgery and an IPC might not be of benefit to them)

   b. Ideally patients should have a WHO PS 0-3

      (Patients with a PS of 4 might be considered- please discuss with senior as an IPC reduces hospital admissions and can be a definitive procedure)

   c. Send email to avinash.aujayeb@nhct.nhs.uk, sarah.robinson@nhct.nhs.uk, leonie.armstrong@nhct.nhct.uk

Grade	Explanation of Activity
0	Fully active, able to carry on all pre-disease performance without restriction
1	Restricted in physically strenuous activity but ambulatory and able to carry out work of a light or sedentary nature, e.g., light house work, office work
2	Ambulatory and capable of all self-care but unable to carry out any work activities. Up and about more than 50% of waking hours
3	Capable of only limited self-care, confined to bed or chair more than 50% of waking hours
4	Completely disabled. Cannot carry on any self-care. Totally confined to bed or chair

2. **Explanation to patient**

   a. The IPC is a silicone tube which goes through the skin and into the abdomen. It is semi-permanent and can be easily connected to bag to allow drainage of ascites without any further surgical procedure.

      This prevents hospital admissions and is cost effective. It allows drainage of the ascites in the patient’s own home.

      The IPC is being offered as an alternative to repeated paracentesis.

Symptomatic Malignant Ascites

- Refer for USS if appropriate (i.e. if definitive intervention appropriate/possible)
- Significant ascites on USS amenable to drainage
  - No → Inform referring person to get back in touch when ascites has increased
  - Yes → Previous ascitic drain?
    - No → Refer for paracentesis in Ambulatory care
    - Yes → Assess performance status (See above re gynae malignancies)
      - PS 4 → Discuss with senior or specialist (contact details above) to consider if IPC still appropriate? If not: either; Make plans for repeated paracentesis in Ambulatory care Or Manage symptoms using alternative method (analgesia etc)
      - PS 1,2,3 → Refer for IPC
        - Patient able to wait for IPC?
          - No → Insert ascitic drain for symptomatic relief whilst awaiting IPC
          - Yes → Refer for IPC

Antibiotics required for IPC insertion
1 IV stat dose prior to procedure
<65 Co-amoxiclav
≥65 Tazocin

In all cases consider if referral to palliative care required

References

- Therapeutic Procedures for Malignant Ascites in a Palliative Care Outpatient Clinic Sade Korpi,Veera V. Salminen, Reetta P. Piili, Niina Paunu, Tiina Luukkaala, and Juho T. Lehto Journal Of Palliative Medicine 2018
- NICE Guidance PleurX peritoneal catheter drainage for treatment-resistant, recurrent ascites MTG 9

Further information/contacts

…………………Avinash.Aujayeb@nhct.nhs.uk………………………………..

Add to Intranet – Please Indicate appropriate Specialties:

: Acute and Emergency Medicine, Gastroenterology and General Surgery
